# TRANSITIONS BETWEEN MEDICARE FEE-FOR-SERVICE AND MEDICARE ADVANTAGE AMONG OLDER ADULTS WITH CANCER

**DOI:** 10.1093/geroni/igae098.2405

**Published:** 2024-12-31

**Authors:** Maira Castañeda-Avila, Mara Epstein, Kate Lapane

**Affiliations:** University of Massachusetts Chan Medical School, Worcester, Massachusetts, United States; University of Massachusetts Chan Medical School, Worcester, Massachusetts, United States; University of Massachusetts Chan Medical School, Worcester, Massachusetts, United States

## Abstract

To date, SEER-Medicare analyses focused on Medicare Fee for Service (FFS) patients due to data availability. The rising enrollment in Medicare Advantage (MA) underscores the necessity of including these patients in cancer research. We examined transitions between FFS and MA among older adults diagnosed with cancer and explored factors associated with coverage by FFS, MA, or transitions between these types of insurance. From the time of diagnosis to end of follow-up, 62.3% were continuously enrolled in FFS, 21.3% were continuously enrolled in MA, 11.8% transitioned from MA to FFS, and 4.5% transitioned from FFS to MA. Continuous enrollment in MA was more common among women (Odds Ratio (OR) =1.25, 95% Confidence Interval (CI):1.22-1.28), Hispanic (OR=2.21, 95%CI: 2.14-2.28) and Black adults (OR=1.79, 95% CI: 1.74-1.85) and less likely among older adults ≥74 (OR=0.85, 95%CI: 0.83-0.86), unmarried people (OR=0.85, 95%CI:0.83-0.87), and people with brain, lung, and ovary cancer relative to prostate cancer (OR ranges: 0.35-0.46). Factors associated with transitioning from MA to FFS after a cancer diagnosis included age ≥74 (OR: 1.82; 95%CI: 1.77-1.86), Hispanic (OR: 1.98; 95%CI: 1.91-2.60) and Black adults (OR=1.56, 95%CI:1.91-2.06), or having a diagnosis of colorectal, leukemia, lung, lymphoma, multiple myeloma, oral cavity, ovary, and brain cancer relative to prostate cancer (OR range: 1.96-3.69). Black (OR=2.20, 95%CI:2.09-2.33) and Hispanic (OR=1.70, 95%CI:1.60-2.33) adults were more likely to transition from FFS to MA. Understanding reasons for switching types of Medicare insurance among patients with cancer may be important for interpreting studies of social determinants of health using SEER-Medicare data.

